# Athlete’s heart in a Brazilian paralympic judo team. Case series study

**DOI:** 10.1590/1516-3180.2017.0240281017

**Published:** 2018-01-09

**Authors:** Japy Angelini Oliveira, Maria Beatriz Monteiro Barros, Ana Fátima Salles, Leandro Santini Echenique, Orlando Campos, Rui Manoel Santos Póvoa

**Affiliations:** I MD, PhD. Associate Professor, Cardiology Division, Department of Medicine, Escola Paulista de Medicina - Universidade Federal de São Paulo (EPM-Unifesp), São Paulo (SP), Brazil.; II MD. Attending Physician, Sports Medicine Division, Department of Orthopedics and Traumatology, Escola Paulista de Medicina - Universidade Federal de São Paulo (EPM-Unifesp), São Paulo (SP), Brazil.; III MD. Attending Physician, Cardiology Division, Department of Medicine, Escola Paulista de Medicina - Universidade Federal de São Paulo (EPM-Unifesp), São Paulo (SP), Brazil.; IV MD. Attending Physician, Cardiology Division, Department of Medicine, Escola Paulista de Medicina - Universidade Federal de São Paulo (EPM-Unifesp), São Paulo (SP), Brazil.; V MD, PhD. Adjunct Professor, Cardiology Division, Department of Medicine, Escola Paulista de Medicina - Universidade Federal de São Paulo (EPM-Unifesp), São Paulo (SP), Brazil.; VI MD, PhD. Adjunct Professor, Cardiology Division, Department of Medicine, Escola Paulista de Medicina - Universidade Federal de São Paulo (EPM-Unifesp), São Paulo (SP), Brazil.

**Keywords:** Exercise, Exercise test, Echocardiography

## Abstract

**BACKGROUND:**

Athlete’s heart is a term describing the cardiovascular effects of long-term conditioning among highly trained athletes. It is a variation of normal standards.

**DESIGN AND SETTING:**

Case series study at the cardiology division of a public university hospital.

**METHODS:**

We studied 14 visually handicapped paralympic athletes (8 men) in the national judo team. They were 26.3 ± 6.4 years old, with body mass index 25 ± 14, and had been practicing judo for 9.2 ± 7.9 years. Clinical evaluations, electrocardiograms, exercise testing and echocardiograms were performed by independent observers.

**RESULTS:**

Signs of athlete’s heart were found in all athletes, comprising left ventricular hypertrophy (5 cases), sinus bradycardia (5), T-wave juvenile pattern (3), T wave juvenile pattern (3), left atrial hypertrophy (2) and increased left ventricular volume (9 cases; 62.22 ± 6.46 ml/m^2^). There were very strong correlations between left ventricular mass/body surface and endurance time (r: 0.91) and estimated peak oxygen uptake (r: 0.8). The correlations between left ventricular internal diastolic dimension and endurance time (r: 0.91) and estimated peak oxygen uptake (r: 0.8) were strong. Despite increased left ventricular dimensions (4 cases), atrial dimensions (1) and relative wall thickness (4), all athletes had normal left ventricular mass/body surface (89.98 ± 21.93 g/m²). The exercise testing was normal: exercise duration 706 ± 45 seconds and estimated peak oxygen uptake 62.70 ± 9.99 mlO_2_/min.

**CONCLUSIONS:**

Signs of athlete’s heart were seen frequently in the paralympic judo team. These demonstrated the presence of mild cardiac adaptations to training.

## INTRODUCTION

Athlete’s heart is a term used to describe the cardiovascular effects of long-term conditioning that are observed among highly trained athletes.^1^ The first report was made by Henschen among Swedish skiers in 1899.^2^ This condition includes clinical, electrocardiographic and echocardiographic signs and the prognostic implications are good.^3^^,^^4^ It gives rise to increased left ventricular dimensions, as a cardiovascular adaptation to long-term athletic training. It also frequently enlarges the wall thickness and the mass of the heart.^5^^,^^6^

Athlete’s heart is a variation of normal standards. Occurrences of cardiac adaptations to training among disabled athletes have been already observed. Among Brazilian elite disabled athletes, signs of athlete’s heart have been found to occur in 33% of clinical evaluations, 55% of electrocardiograms, 15% of vectorcardiograms and 5% of echocardiograms.^7^

At least one of these signs has been found to be presented by 51% of disabled athletes.^7^ These individuals were found to have reasonably high prevalence of coronary risk factors (51%), despite a low likelihood of coronary events.^8^

The aim of the present case series study was to assess occurrences of athlete’s heart among the Brazilian paralympic judo team.

## METHODS

We studied the entire national paralympic judo team, comprising 14 athletes, who were all visually handicapped. Eight of them were men. The paralympic athletes were 26.3 ± 6.4 years old, with body mass index (BMI) = 25 ± 14 kg/m^2^, and had been practicing judo for 9.2 ± 7.9 years. Clinical evaluations, electrocardiograms (ECG), exercise testing and echocardiograms were performed by independent observers. Examinations were performed during periods of peak training. ECG evaluations followed the third guidelines of the Brazilian Society of Cardiology regarding analysis and issuing of electrocardiographic reports and the criteria of Corrado et al. for diagnosing athlete’s heart.^9^

All the subjects underwent symptom-limited evaluations on a treadmill (TM48 Trackmaster, JAS System, Pensacola, Florida, USA), in accordance with the Bruce protocol (TEB, Apex 2000 System, São Paulo, Brazil). Echocardiograms were recorded on the ATL Ultramark 8 and 9 devices (Bothell, WA, USA), using a 3.0-MHz phased-array transducer.

We evaluated left ventricular volume (LVV), left ventricular mass/body surface (LVM/BS), relative wall thickness (RWT), diastolic interventricular septum thickness (IVSTd), diastolic posterior left ventricular wall thickness (PLVWTd), left ventricular internal diastolic dimension (LVIDd), left ventricular ejection fraction (LVEF), percentage of fractional shortening (PFS), left atrial dimension (LAD) and right ventricular end diastolic inner diameter (RV-EDD).^10^

Prior to the evaluation, informed consent was obtained from each patient. The study protocol conformed with the ethical guidelines of the 1975 Declaration of Helsinki, as reflected through a priori approval from our institution’s human research committee (CAAE: 62709816.2.0000.5505).

Pearson correlation coefficients (r) were used to estimate relationships between the exercise test results and echocardiographic variables. The significance level was taken to be P < 0.05. All data were expressed as mean ± standard deviation (SD).

## RESULTS

The results are described in Tables 1 and 2. The clinical evaluation showed that most of the paralympic athletes (n = 12) were asymptomatic and apparently healthy (n = 10). One subject showed obesity, asthma and mild arterial hypertension; another presented obesity and two others had asthma. Systolic mild cardiac murmurs were detected in two paralympic athletes.

**Table 1:** Clinical, electrocardiographic and echocardiographic findings among athletes in the Brazilian paralympic judo team

Data	n
Age (years)	26.3 ± 6.4
Men	8 (57%)
Women	6 (43%)
Clinical findings	
Visually handicapped	14 (100%)
Systolic murmurs	2
Electrocardiogram	
Sinus rhythm	15
Nonspecific interventricular conduction defect	7
Left ventricular hypertrophy	5
Sinus bradycardia	3
T wave juvenile pattern	3
Left atrial hypertrophy	2
Atrioventricular block, first degree	1
Echocardiogram	
Increased left ventricular volume	9
Increased left ventricular internal diastolic dimension	4
Increased relative wall thickness	4
Increased interventricular septum thickness	1
Increased interventricular septum thickness	1
Variables	Mean ± standard deviation
Exercise testing	
Rest heart rate (bpm)	66 ± 4
Exercise time (sec)	706.35 ± 44.54
Estimated peak oxygen uptake (mlO_2_/minute)	62.70 ± 9.99
Echocardiogram	
Left ventricular volume (g/m²)	62.22 ± 6.46
Left ventricular mass/body surface (g/m²)	87.98 ± 21.93
Relative wall thickness (g/m²)	0.39 ± 0.04
Interventricular septum thickness (mm)	8.92 ± 1.53
Posterior left ventricular wall thickness (mm)	9.57 ± 1.22
Left ventricular internal diastolic dimension (mm)	49.46 ± 1.41
Left ventricular ejection fraction	0.66 ± 0.29
Percentage of fractional shortening (%)	36.29 ± 2.12
Left atrial dimension (mm)	38.07 ± 1.41
Right ventricular end diastolic inner diameter (mm)	20.71 ± 1.41

**Table 2:** Correlations between endurance time and echocardiographic variables among 14 paralympic judo players

Variable	Pearson’s coefficient
Left ventricular mass/body surface	0.91
Left ventricular internal diastolic dimension	0.91
Interventricular septum thickness	0.48
Posterior left ventricular wall thickness	0.42
Right ventricular end diastolic inner diameter	0.21
Left atrial dimension	0.03

The left ventricular volume was increased in nine athletes (62.22 ± 6.46 ml/m^2^), ranging from 52 ml/m^2^ to 95 ml/m^2^. Despite the increased left ventricular dimensions (n = 4), atrial dimensions (n = 1) and relative wall thickness (n = 4), all the athletes had normal left ventricular mass/body surface (89.98 ± 21.93 g/m²). The right ventricular dimensions were also within normal values. The ejection fraction (66.23 ± 2.94) and the percentage of fractional shortening (36.29 ± 2.18%) were also normal. The Doppler echocardiography did not detect any significant valvular regurgitant flow.

The results from exercise testing were normal for all subjects. There were no cases of ischemic ST depressions on ECG, or any cases of arrhythmias or hypotension. In the exercise testing, the exercise duration was 706 ± 45 seconds and the estimated peak oxygen uptake reached 62.70 ± 9.99 mlO_2_/minute. According to the criteria of the American Heart Association, the physical fitness was excellent (in 7% of the cases), good (36%), regular (21%) and weak (7%). This was not assessed in 29% of the cases because these individuals were under 20 years of age.^11^ Signs of athlete’s heart were found in 100% of the disabled athletes.

The correlations between the variables are presented in Table 2. There were very strong correlations between left ventricular mass/body surface (LVM/BS) and endurance time (r = 0.91) and estimated peak oxygen uptake (r = 0.8). The correlations between left ventricular internal diastolic dimension (LVIDd) and endurance time (r = 0.91) and estimated peak oxygen uptake (r = 0.8) were also strong.

## DISCUSSION

We studied the Brazilian paralympic judo team to assess occurrences of althlete’s heart. All the subjects were apparently health subjects and performed athletic activities national level. One subject presented obesity (BMI = 36.8 kg/m^2^), slight asthma and mild recent hypertension; one had obesity (BMI = 38.5 kg/m^2^); and another two had mild asthma. All the other subjects had BMI ranging from 20.4 to 28.4 kg/m^2^. These data probably did not interfere with the myocardial findings.

During judo training and competitions, a high static component is required, comprising more than 50% of the estimated percentage of maximal voluntary contraction; and a low dynamic component comprising less than 40% of the estimated percentage of maximal oxygen uptake.^12^ Strength training results in marked elevations in systolic and diastolic blood pressure. It induces large sudden pressure overloads and concentric left ventricular hypertrophy. Sometimes, it increases the left ventricular diameter.^13^

The maximal oxygen uptake in elite judo players has ranged from 45 ± 10 mlO_2_/kg/minute (Germany, 1971) to 59.62 mlO_2_/mg/min^14^^,^^15^ Our athletes reached an excellent estimated oxygen uptake level (62.70 ± 9.99 mlO_2_/kg/min). For 60% of our athletes, their fitness level was considered to be good/excellent.

Aerobic power and capacity levels have been found to be similar between Brazilian elite and non-elite judo players. VO_2_max did not differ (P > 0.05) between the groups: elite (VO_2_max = 58.13 ± 10.83 mlO_2_/kg/min) versus non-elite (VO_2_max = 63.28 ± 10.55 mlO_2_/kg/min).^16^ Despite occurrences of lower aerobic capacity among other paralympic athletes, the exercise duration according to the Bruce protocol among our athletes was 706.35 ± 44.54 seconds and the estimated peak oxygen uptake was 62.70 ± 9.99 mlO_2_/kg/minute.

We found ECG abnormalities in nine athletes and increased echocardiographic measurements in six athletes. Nine subjects presented increased left ventricular volume, although there were no increases in right ventricular end diastolic inner diameter. This measurement may differentiate between normal hearts and exercise-related right ventricular adaptations and is the only parameter recommended for measurement within athletes’ routine training.^14^

We registered very strong correlations between left ventricular mass/body surface and endurance time, thus showing the relationship between training and left ventricular hypertrophy, considering that judo is a martial art involving a high static component. There were also strong correlations between the left ventricular internal diastolic dimension of estimated peak oxygen uptake, considering that judo is also a sport involving a high dynamic component.^17^

Although intense endurance exercise may cause acute dysfunction of the right ventricle, chronic structural changes and reduced levels of right ventricle function in some athletes^18^, we did not find any correlations between right ventricle dimensions and functional variables. The long-term clinical significance of these data warrants further study.^18^

Athlete’s heart is a term used to describe the cardiovascular effects of long-term conditioning that is observed in highly trained athletes.^1^ In our study, nine athletes showed ECG signs of athlete’s heart and six athletes presented left ventricular hypertrophy on echocardiograms. Among all the subjects, eleven individuals (79%) were assessed as presenting athlete’s heart. These data must be considered in clinical evaluations and management.

## CONCLUSION

Signs of athlete’s heart were highly prevalent manifestations among these paralympic judo players. This demonstrated that these disabled athletes presented mild cardiac adaptations to training.
